# Purification and Identification of Antioxidant Peptides from *Schizochytrium Limacinum* Hydrolysates by Consecutive Chromatography and Electrospray Ionization-Mass Spectrometry

**DOI:** 10.3390/molecules24163004

**Published:** 2019-08-19

**Authors:** Xiao Hu, Xianqing Yang, Qiong Wu, Laihao Li, Yanyan Wu, Shengjun Chen, Ruijie Li, Jiaoyan Ren

**Affiliations:** 1 Key Laboratory of Aquatic Product Processing, Ministry of Agriculture and Rural Affairs, South China Sea Fisheries Research Institute, Chinese Academy of Fishery Sciences, Guangzhou 510300, China; 2 Co-Innovation Center of Jiangsu Marine Bio-industry Technology, Jiangsu Ocean University, Lianyungang 222005, China; 3 College of Food Science and Engineering, Ocean University of China, Qingdao 266003, China; 4 School of Food Sciences and Engineering, South China University of Technology, Guangzhou 510641, China

**Keywords:** *Schizochytrium limacinum* hydrolysates, antioxidant peptides, radical scavenging activity, reducing power, electrospray ionization-mass spectrometry

## Abstract

*Schizochytrium limacinum* residue was hydrolyzed with various proteases (papain, trypsin, Flavourzyme, Protamex, and Alcalase 2.4L) to obtain antioxidative peptides. The results showed that the *S. limacinum* hydrolysates (SLHs) prepared with compound proteases (Protamex and Alcalase 2.4L) had the highest antioxidant activity, which was measured using methods such as 1,1-diphenyl-2-picrylhydrazyl (DPPH) radical scavenging ability (IC_50_ = 1.28 mg/mL), hydroxyl radical scavenging ability (IC_50_ = 1.66 mg/mL), and reducing power (1.42 at 5.0 mg/mL). The hydrolysates were isolated and purified by ultrafiltration, gel filtration chromatography, and reverse-phase high-performance liquid chromatography (RP-HPLC). Through analysis of electrospray ionization-mass spectrometer (ESI-MS/MS), the purified antioxidant peptide was identified as Pro-Tyr-Lys (406 Da). Finally, the identified peptide was synthesized for evaluating its antioxidant activity. The •OH scavenging ability and reducing power of Pro-Tyr-Lys were comparable to those of reduced L-glutathione (GSH). These results demonstrated that the antioxidant peptides from SLHs could potentially be used as effective antioxidants.

## 1. Introduction

Food-derived bioactive peptides, with specific amino acid sequence and biological activities, are released from proteins by protease hydrolysis. Antioxidant peptides, as a kind of active peptide, could remove free radicals in the human body to reduce lipid, protein, and DNA oxidative degradation and oxidative damage in cells [1]. Moreover, these peptides also help in preventing many chronic diseases such as diabetes, cancer, and cardiovascular diseases [2]. 

In recent years, research interest in natural sources of antioxidants has grown substantially, due to their wide distribution, coupled with their low molecular weight and strong antioxidative activities. The antioxidant peptides prepared from aquatic protein hydrolysates, mainly from fish, shrimp, shellfish, and seaweed sources, have received great attention [3,4,5,6]. Marine microalgae, an important natural marine biological resource, are rich in polyunsaturated fatty acids and a variety of bioactive substances such as polysaccharides and peptides. However, until now, they are mainly used for the preparation of biological energy and aquaculture feed, and limited research has been done for their preparation as an antioxidative substance [7,8,9].

*Schizochytrium limacinum* is a kind of microalgae that is rich in polyunsaturated fatty acids, and it is mostly used to produce healthcare products such as DHA-rich oil and infant dairy additives. *S. limacinum* residue is a by-product obtained from oil extraction. This underutilized microalgae residue, containing more than 40% protein, has a low economic value being used as animal feed or fertilizer. Recently, researchers have found that microalgae contains many kinds of bioactive components (e.g., phycoerythrin, phycocyanin, and allophycocyanin), and the aqueous extracts of microalgae can exhibit various bioactivities (e.g., antioxidant and anticancer activities) [10,11]. It is hypothesized that *S. limacinum* hydrolysates (SLHs) could have antioxidant activity as well. However, little information is available on the antioxidant properties of the peptides derived from *S. limacinum*.

In the present study, the antioxidant activity of SLHs obtained from *S. limacinum* residue by different proteases hydrolysis was investigated, and its antioxidant mechanism was explored by measuring 1,1-diphenyl-2-picrylhydrazyl (DPPH) radical scavenging activity, hydroxyl radical scavenging ability, and reducing power. Moreover, an antioxidant peptide was isolated from the hydrolysates and purified by ultrafiltration, gel filtration chromatography, and reverse-phase high-performance liquid chromatography (RP-HPLC). The sequence of the peptide was identified using an electrospray ionization-mass spectrometer (ESI-MS/MS). Finally, the identified peptide was synthesized for determining its antioxidant activity.

## 2. Results and Discussion

### 2.1. Antioxidant Activity of SLHs

SLHs obtained by five proteases including papain, trypsin, Flavourzyme, Protamex, and Alcalase 2.4L, were tested for their antioxidant activities including DPPH scavenging activity, •OH scavenging activity, and reducing power assays. Flavourzyme, Protamex, and Alcalase 2.4L were found to be more efficient than the others, and the resulting hydrolysates exhibited higher antioxidant capacity (Table 1). Therefore, these three proteases were selected for further hydrolysis.

Different combinations of these proteases were also applied in order to obtain hydrolysates with better antioxidant activities. As shown in Table 1, the application of a combination of two or more proteases enhanced bioactivity, indicated by stronger activities compared to those from single protease. Moreover, the combination of Alcalase 2.4L and Protamex was proven to be most effective and demonstrated the most potent DPPH radical scavenging ability among all the composite methods (IC_50_ = 1.28 mg/mL). It addition, it also exhibited strong •OH scavenging and reducing power activities. Taken together, it could be said that the Alcalase 2.4L–Protamex combination produced the hydrolysates with best antioxidant activity. The SLHs made from the combination of Protamex and Alcalase 2.4L were then further isolated for the identification of antioxidant peptides.

### 2.2. Isolation and Purification of Antioxidant Peptides

#### 2.2.1. Ultrafiltration

Ultrafiltration membranes (molecular weight cutoff (MWCO) = 50, 10, and 5 kDa) were used to separate the hydrolysates made from Protamex–Alcalase 2.4L combination into three fractions, SLH-I (<50 kDa), SLH-II (<10 kDa), and SLH-III (<5 kDa). As shown in Table 2, SLH-III fraction exhibited the strongest DPPH (65.50%) and reducing power activities (0.48), which were significantly higher (*p* < 0.05) than those of the other fractions. This result is consistence with that of Ren et al. [12], who also found that low-molecular-weight (Mw) peptides had higher antioxidant activities than higher Mw fractions. Therefore, the fraction Mw < 5 kDa was selected for isolation and further purification for antioxidant peptides.

#### 2.2.2. Gel Filtration Chromatography

**Figure 1:**
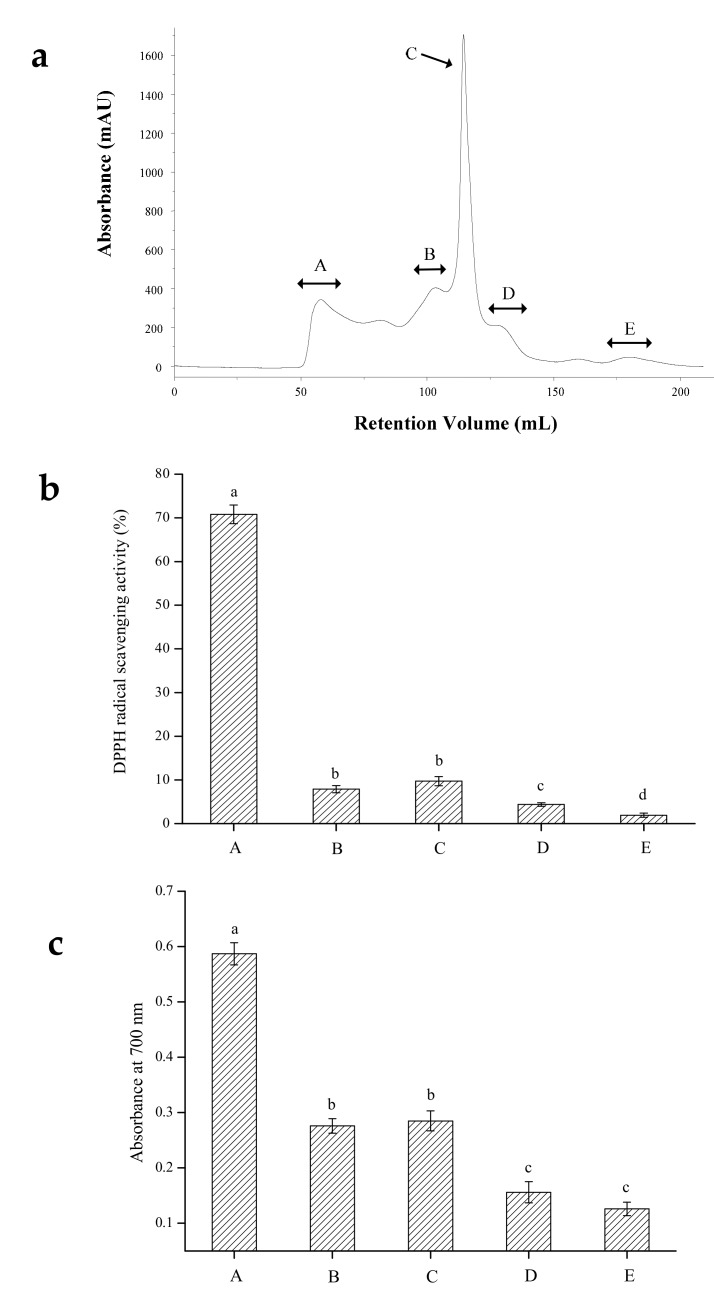
(**a**) Chromatogram of fraction SLH-III purified by gel filtration column; (**b**) DPPH scavenging ability of each fraction (samples at 0.5 mg/mL); (**c**) reducing power of each fraction (samples at 1 mg/mL). The columns having the same letter are not significantly different (*p* > 0.05).

Gel filtration chromatography has been used for group separation of biological extracts and protein hydrolysates. Gel filtration chromatography is a method for separation of substances with different molecular weight and has also been applied for desalting protein solutions [12]. SLH- III was further separated into five fractions (A–E) by gel filtration chromatography on a Sephadex G-25 column (Figure 1a). Each fraction was pooled, lyophilized, and tested for antioxidant activity including DPPH scavenging ability and reducing power. As shown in Figure 1b,c, fraction A exhibited the strongest DPPH radical scavenging activity with a rate of 70.80% at 0.5 mg/mL and the highest reducing power of 0.587 at 1 mg/mL. The fraction with shorter retention time may have larger molecular weight since gel filtration chromatography separates peptides based on their molecular weight [13]. The antioxidant activity of peptides is considered to be related to their molecular weight and chain length [2,14]. Although some research findings indicated that small molecular peptides possess stronger antioxidant activities, others suggest that peptides with larger molecular weight had higher antioxidant activity [15]. In this research, we found that the antioxidant activity of the eluted fractions was weakened with the increasing retention time. Consequently, the fraction SLH-III-A, which demonstrated the strongest activity, was selected for further analysis.

#### 2.2.3. RP-HPLC Purification

**Figure 2:**
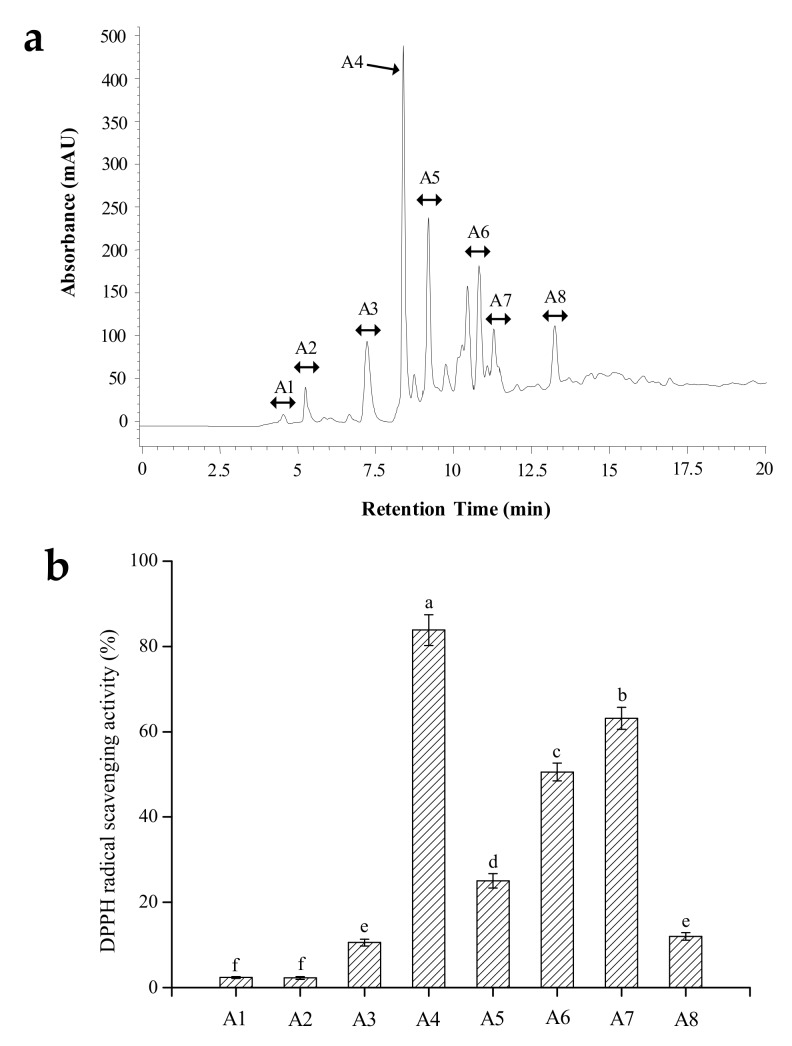
(**a**) Reverse-phase high-performance liquid chromatography (RP-HPLC) separation of the fraction A obtained after gel filtration chromatography; (**b**) DPPH scavenging ability of each fraction (samples at 0.5 mg/mL). The columns having the same letter are not significantly different (*p* > 0.05).

The active fraction SLH-III-A was then separated by RP-HPLC using a semi-preparative ZORBAX SB-C18 column (9.4 × 250 mm, 5 μm, Agilent Technology, Santa Clara, CA, USA). A total of eight fractions (A1–A8) were collected (Figure 2a). Among these eight fractions, portion A4 (SLH-III-A4) showed the strongest DPPH scavenging activity of 83.84% at 0.5 mg/mL, which is about 3.2-fold higher than that of the crude SLH sample (Table 2 and Figure 2b). According to the property of C18 column, the eluted order of each fraction peak was correlated to the polarity of samples. Generally, the hydrophobic components stay firmly within the column and are harder to be eluted out. The fraction SLH-III-A4 was eluted within a relatively shorter retention time, indicating that it may contain polar amino acids [16]. The structural characteristics of SLH-III-A4 were further identified.

### 2.3. Identification of Antioxidant Peptides by ESI-MS/MS

**Figure 3:**
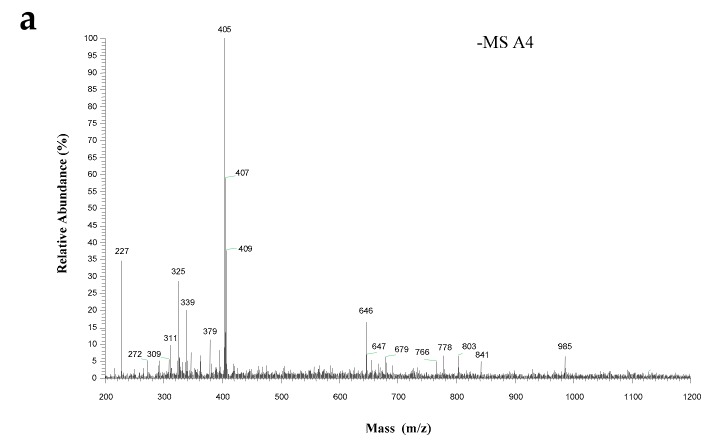

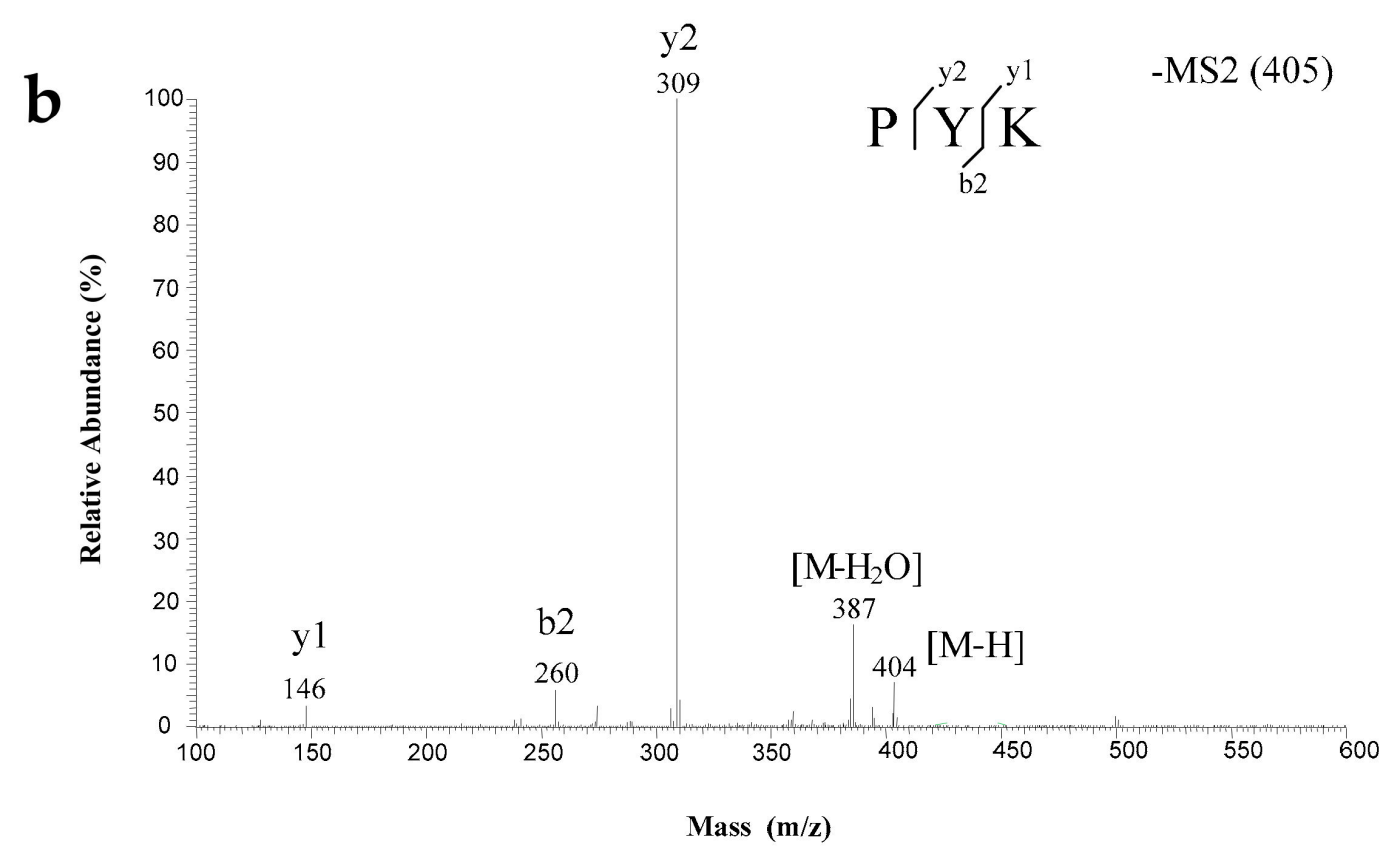
(**a**) Mass spectrum of the chromatographic peak A4 in Figure 2a; (**b**) MS/MS spectrum of ion *m*/*z* 405.

Mass spectrometric approaches have been widely used for peptide sequence identification due to their high sensitivity [17]. To identify the main antioxidant peptides responsible for the observed activity, SLH-III-A4 fraction was further sequenced by ESI-MS/MS. The MS spectrum of this fraction is shown in Figure 3a and the MS/MS spectrum of a single charged ion with *m*/*z* at 405 Da is illustrated in Figure 3b. The molecular mass of the antioxidant peptide was determined to be 406 Da. Since each single peptide fragment matches to specific mass number corresponding fragmentation spectra [18], by combining the MS data with manual calculations and data search, the amino acid sequence of the targeted peptide in this study was identified to be Pro-Tyr-Lys.

According to the naming system proposed by Roepstorff and modified by Biemann [19], N-terminal fragment ions were expressed with the letters a, b, and c and C-terminal fragment ions were expressed with the letters x, y, and z. Because the amide bond is relatively easily broken in peptides, b-type and y-series fragments may appear more frequently in the mass spectrum. Fragments of y2 (*m*/*z* 309), b2 (*m*/*z* 260), and y1 (*m*/*z* 146) were all generated by peptide bond cleavage in this paper. Moreover, the fragments may also lose a neutral molecule of water or ammonia [20], resulting in the high abundance of *m*/*z* 387.

**Figure 4:**
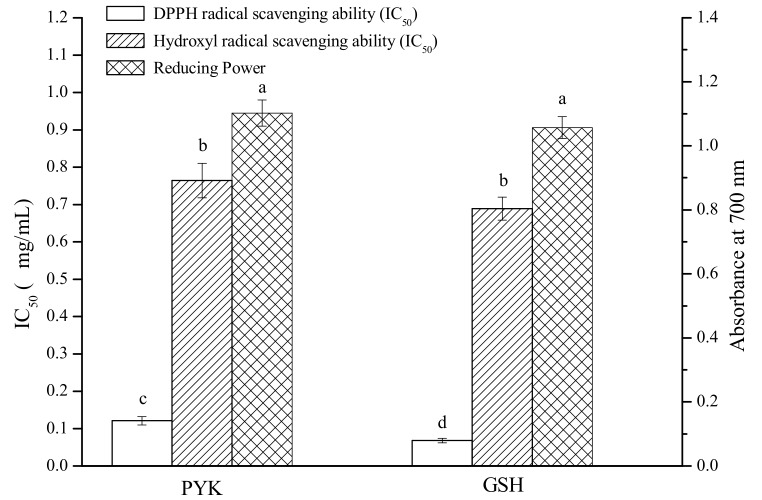
Radical scavenging ability (IC_50_) and reducing power (samples at 0.5 mg/mL) of Pro-Tyr-Lys (PYK) and L-glutathione GSH. The columns having the same letter are not significantly different (*p* > 0.05).

The identified peptide with the amino acid sequence of Pro-Tyr-Lys (PYK) was synthesized for evaluating its antioxidant activity. The radical scavenging ability and reducing power of PYK were determined, which were compared with that of reduced L-glutathione (GSH). As shown in Figure 4, although PYK had a higher DPPH· scavenging IC_50_ value (IC_50_ = 0.12 mg/mL) compared to GSH (IC_50_ = 0.07 mg/mL), the •OH scavenging ability and reducing power of PYK were not significantly different from that of GSH (*p* > 0.05). This result demonstrated that PYK, which was identified from antioxidative peptides in SLHs, possessed excellent antioxidant activities.

Research shows that peptides with 2–10 amino acids exhibited greater antioxidant activity than larger polypeptides and some active peptides were capable of inhibiting lipid peroxidation in food systems and sequestering oxygen radicals [21,22]. The antioxidant activities of di-, tri-, and tetra-peptides were shown to be comparable to ascorbic acid at 0.1 mg/mL or butylated hydroxyanisole (BHA) [23]. The antioxidant activities of peptides are also attributed to the effects of the constituent and sequence of the amino acid [24]. It is reported that residues like Pro, Met, Tyr, Phe, and Trp enhance the antioxidant activities [25]. The identified peptide in this study contained two hydrophobic amino acids, Pro and Tyr, and these may have contributed to its stronger antioxidant activities. Furthermore, Tyr as an aromatic amino acid had been shown to act as a direct radical scavenger [26]. Research findings involving synthetic peptides showed that reducing the Tyr number in a peptide sequence affected the antioxidant capacity of the peptides, and the loss of even one Tyr could lead to significant decrease in antioxidant capacity [27,28]. In addition, Lys contributes to the termination of radical chain reaction, the interaction with free radicals, and the prohibition of radical formation. Moreover, relevant research confirmed that basic amino acid (Lys) in peptides could be an acceptor of electron, accepting the free radicals produced from the oxidation of unsaturated fatty acids [29]. All these findings further implied that the identified tripeptide, Pro-Tyr-Lys, could be used as an effective antioxidant.

## 3. Materials and Methods

### 3.1. Materials and Chemicals

*S. limacinum* residue was obtained from Guangdong Runke Bioengineering Co., Ltd. (Shantou, China). The residue was packed in vacuum during transportation and stored at 4 °C for use. Five food-grade enzymes (papain, trypsin, Flavourzyme, Protamex, and Alcalase 2.4L) were purchased from Guangzhou Qiyun Biological Technology Co., Ltd. (Guangzhou, China) and Novozymes Biotechnology Co., Ltd. (Tianjin, China). 1, 1-diphenyl-2-picrylhydrazyl (DPPH) and 1, 10-phenanthroline were obtained from Sigma-Aldrich (St. Louis, MO, USA). Reduced l-glutathione (GSH) was purchased from Hefei Bomei Biotechnology Co., Ltd. (Hefei, China). All other chemicals and reagents were of analytical grade.

### 3.2. Preparation of S. Limacinum Hydrolysates (SLHs)

*S. limacinum* residue (100 g) was mixed with 1200 mL of distilled water. Single commercial protease (e.g. papain, trypsin, Flavourzyme, Protamex, and Alcalase 2.4L) as well as different combinations of these proteases (shown in Table 1) were tested. The total enzyme to substrate ratio (E/S) was 1:200 (w/w). The hydrolysis was conducted for 6 h in a water bath shaker. At the end of the hydrolysis, the mixtures were heated in boiling water for 15 min to inactivate the proteases, and then centrifuged at 10,000× *g* for 10 min at 4 °C (3K30 refrigerated centrifuge, Sigma, Osterode, Germany) to eliminate the sediment. The supernatants were collected, lyophilized, and stored at −18 °C.

### 3.3. Determination of Antioxidant Activities of SLHs

#### 3.3.1. DPPH Radical Scavenging Activity Assay

The scavenging activity of DPPH radical was assayed according to the method of Gu et al. [30] with some modifications. Sample (0.5 mL) was mixed thoroughly with 0.5 mL of DPPH solution (0.2 mM, dissolved in ethanol) and kept in the dark for 20 min. The mixture was centrifuged at 10,000× *g* for 10 min. Then the absorbance was measured at 517 nm by a microplate reader (Sunrise-basic TACAN, Mannedorf, Switzerland). Ethanol solvent instead of DPPH solution was used as blank, and ethanol instead of sample was used for the control. The scavenging activity of DPPH radical was calculated by the following equation:

DPPH radical scavenging activity (%) = [1 − (A_i_ − A_j_)/A_0_] × 100%, (1)

where A_i_, A_j_, and A_0_ were the absorbances of the sample, blank, and control, respectively. The IC_50_ values (concentration of samples to decrease 50% of scavenging activity) were plotted against the concentrations of individual samples.

#### 3.3.2. Hydroxyl Radical (•OH) Scavenging Activity Assay

Hydroxyl radical (•OH) scavenging activity was determined by the method of Ajibola et al. [31] with some modifications. The reaction mixture consisted of 0.3 mL of 1,10-phenanthroline (5 mM, dissolved in ethanol), 0.2 mL of 0.15 M sodium phosphate buffer (pH 7.4), 0.3 mL of 0.75 mM FeSO_4_ and 1 mL of sample. Then, 0.2 mL of hydrogen peroxide (0.1%, w/w) was thoroughly mixed in a tube and kept in a 37 °C water bath for 1 h. After centrifuging at 10,000× *g* for 10 min, the absorbance was measured at 536 nm. Deionized water instead of sample was used as blank, and deionized water instead of hydrogen peroxide was used as control. The •OH scavenging activity was evaluated by the following equation:

•OH scavenging activity (%) = [(A_i_ − A_j_ )/(A_0_ − A_j_)] × 100%, (2)

where A_i_ was the absorbance of sample, A_j_ was the absorbance of blank, and A_0_ was absorbance of the control. •OH scavenging activity values were plotted against the concentrations of individual samples and the IC_50_ was measured.

#### 3.3.3. Reducing Power Assay

Reducing power assay was performed as reported by Ahmadi et al. [32]. Briefly, a mixture of 1 mL sample, 1 mL of potassium ferrocyanide (1%, w/v), and 1 mL of sodium phosphate buffer (0.2 M, pH 6.6) was incubated at 50 °C for 20 min. Then, 1 mL of 10% trichloroacetic acid was added and the obtained mixture was centrifuged at 10,000× *g* for 10 min. After that, 1 mL of the upper layer was collected and mixed with 0.2 mL FeCl_3_ (0.1%, w/v) and 1 mL distilled water. The reagents were stored at 50 °C for 10 min and the absorbance was detected at 700 nm.

### 3.4. Purification of Antioxidant Peptide from SLHs

#### 3.4.1. Ultrafiltration

The *S. limacinum* hydrolysates (SLHs) were dissolved in distilled water and then fractionated through an ultrafiltration membrane system (Labscale tangential-flow filtration system, Milipore, Billerica, MA, USA) with a molecular weight cutoff (MWCO) of 50, 10, and 5 kDa, respectively. Three fractions, SLH-I (<50 kDa), SLH-II (<10 kDa), and SLH-III (<5 kDa) were obtained. All samples were lyophilized and subjected to antioxidant activity test.

#### 3.4.2. Gel Filtration Chromatography

The fraction with the highest antioxidant activity after ultrafiltration was re-dissolved in ultrapure water, then separated with a Sephadex G-25 gel filtration chromatography column (1.6 cm × 78 cm, Amersham Biotech, GE, Piscataway, NJ, USA) using an AKTA Purifier 100 system (Amersham Biotech, GE, Piscataway, NJ, USA). It was eluted with ultrapure water at a flow of 1.0 mL/min. The elution curve was obtained by measuring the absorbance at 280 nm. The fractions with desired peaks were pooled, concentrated, and lyophilized for further antioxidant activity evaluation.

#### 3.4.3. RP-HPLC

The fraction with the highest antioxidant activity after gel filtration chromatography separation was further purified by using RP-HPLC on an ZORBAX SB-C18 column (9.4 × 250 mm, 5 μm, Agilent, Santa Clara, CA, USA), at a flow rate of 2.0 mL/min. After 10 μL of sample (50 mg/mL) was loaded to the system, the column was eluted with a linear gradient solvent for 20 min [A: 0%–15% acetonitrile; B: 100%–85% trifluoroacetic acid (0.05%, *w*/*w*)]. The elution peaks were detected at 280 nm, then pooled, concentrated, and lyophilized for activity test.

### 3.5. Identification of Antioxidant Peptide by ESI-MS/MS

The desirable fraction after gel filtration chromatography and RP-HPLC purification was injected for mass spectrometer (LTQ Orbitrap Elite, Thermo Fisher Scientific, Waltham, MA, USA) assay, which was operated in a negative electrospray ionization (ESI^−^) mode. Spectra were recorded over the mass/charge (*m*/*z*) range 200–2000. The drying and ESI nebulizing gas was high-purity N_2_. The drying heater was set at 200 °C and the voltage of the capillary was 3.0 kV. The peptide sequencing was performed by processing the MS/MS spectra using BioTools (Version 3.0, Bruker Daltonics Inc., Billerica, MA, USA) as well as manual calculation. The identified peptide was synthesized by GL Biochem (Shanghai, China) Ltd. for the analysis of its antioxidant activity, using solution-phase peptide synthesis methods.

### 3.6. Statistical Analysis

The assay for the hydrolysates was conducted with three replicates and the results were presented as means ± standard deviations (SD). Data analyses were performed by one-way analysis of variance (ANOVA) using SPSS 13.0 (SPSS Inc., Chicago, IL, USA). Significant difference was determined using the least significant difference (LSD) range test (*p* < 0.05).

## 4. Conclusions

Antioxidant peptides were firstly obtained from *S. limacinum* residue by proteolytic hydrolysis with Protamex and Alcalase 2.4L. The hydrolysates exhibited strong DPPH scavenging activities, hydroxyl radicals, and reducing power. The membrane ultrafiltration-separated fractions had low molecular weight (Mw < 5 kDa) and possessed better antioxidant capacities. The peptides were subjected to further isolation and purification by gel filtration chromatography and RP-HPLC. The peptide was identified as Pro-Tyr-Lys (406 Da) using ESI-MS/MS. The identified peptide with the amino acid sequence of Pro-Tyr-Lys was synthesized and demonstrated excellent antioxidant activities. The hydrophobic amino acids (Pro and Tyr) and basic amino acid (Lys) in the peptide sequence might be responsible for the great antioxidant activities. These results indicated that peptides obtained from *S. limacinum* residue by proteolysis could be used as potential natural antioxidants.

## Figures and Tables

**Table 1:** Parameters for enzymatic hydrolysis of *Schizochytrium limacinum* by several single or compound proteases and the antioxidant activities of hydrolysates.

Samples	Parameters for Enzymatic Hydrolysis		Antioxidant Activities ^1^ (IC_50_)(mg/mL)		
	pH	Temperature (°C)	DPPH ^2^	•OH ^3^	Reducing Power ^4^
Papain	6.5	65	1.77 ± 0.09 ^a^	3.12 ± 0.13 ^a^	0.95 ± 0.06 ^f^
Trypsin	8.0	37	1.74 ± 0.11 ^a^	3.29 ± 0.19 ^a^	1.14 ± 0.10 ^e^
Flavourzyme	7.5	50	1.49 ± 0.06 ^b^	2.51 ± 0.02 ^b^	1.20 ± 0.03 ^de^
Protamex	6.5	50	1.46 ± 0.03 ^b^	2.56 ± 0.04 ^b^	1.29 ± 0.03 ^bc^
Alcalase 2.4L	8.0	50	1.55 ± 0.05 ^b^	2.49 ± 0.05 ^b^	1.19 ± 0.02 ^de^
Fla + Pro ^5^	7.5	50	1.45 ± 0.05 ^b^	2.60 ± 0.08 ^b^	1.31 ± 0.04 ^bc^
Fla + Alc ^6^	7.5	50	1.42 ± 0.02 ^b^	1.87 ± 0.02 ^c^	1.37 ± 0.02 ^ab^
Pro + Alc ^7^	7.5	50	1.28 ± 0.03 ^c^	1.66 ± 0.02 ^d^	1.42 ± 0.01 ^a^
Fla + Pro + Alc ^8^	7.5	50	1.54 ± 0.07 ^b^	1.69 ± 0.06 ^cd^	1.26 ± 0.04 ^cd^

^1^ Values within the same column followed by the different letters (a, b, c, d, e and f) are significantly different (*p* < 0.05). ^2^ DPPH radical scavenging ability (DPPH). ^3^ Hydroxyl radical scavenging ability (•OH). ^4^ The samples were at 5 mg/mL. ^5^ Compound of Flavourzyme and Protamex (1:1, w/w). ^6^ Compound of Flavourzyme and Alcalase 2.4L (1:1, w/w). ^7^ Compound of Protamex and Alcalase 2.4L (1:1, w/w); ^8^ Compound of Flavourzyme, Protamex, and Alcalase 2.4L (1:1:1, w/w).

**Table 2:** Antioxidant activity of *S. limacinum* hydrolysates (SLHs) and their fractions from ultrafiltration.

Samples	Molecular Weight (kDa)	Antioxidant Activities ^1^	
		DPPH(%) ^2^	Reducing Power ^3^
SLHs		26.44 ± 2.06 ^b^	0.31 ± 0.01 ^b^
SLH-I	<50	28.03 ± 3.65 ^b^	0.33 ± 0.01 ^b^
SLH-II	<10	31.66 ± 2.83 ^b^	0.38 ± 0.04 ^b^
SLH-III	<5	65.50 ± 4.21 ^a^	0.48 ± 0.03 ^a^

^1^ Values within the same column followed by the different letters (a and b) are significantly different (*p* < 0.05); ^2^ DPPH radical scavenging ability (DPPH), and samples were at 0.5 mg/mL; ^3^ The samples were at 1 mg/mL.
